# Associations among Sleep Quality, Changes in Eating Habits, and Overweight or Obesity after Studying Abroad among International Students in South Korea

**DOI:** 10.3390/nu12072020

**Published:** 2020-07-07

**Authors:** Miae Doo, Chunyang Wang

**Affiliations:** Department of Food and nutrition, Kunsan National University, Daehak-ro 558, Gunsan 54150, Korea; yangyangyang0302@gmail.com

**Keywords:** eating habits, international students, overweight or obesity, sleep quality

## Abstract

International students are experiencing health problems due to many lifestyle changes, such as those in dietary and sleep patterns. We conducted this study to identify the associations among sleep patterns, changes in eating habits after studying abroad, and overweight or obesity in international students. In this cross-sectional study, we analyzed data on health-related variables, changes in eating habits after studying abroad, and sleep patterns that were collected from 225 international students in South Korea. Approximately half of the participants experienced poor sleep (47.6%). After adjusting for covariates such as age, gender, nationality, and acculturative stress, the subjects who had poor sleep quality were 2.020-fold (adjusted odds ratio, 95% confidence interval = 1.045–3.906) more likely to be overweight and obese than those who had good sleep quality. There were significant differences in changes of eating habits after studying abroad according to sleep quality (*p* < 0.001). When subjects were stratified into groups according to changes in eating habits after studying abroad, the risk of overweight and obesity increased in those with poor sleep quality but not in those with good sleep quality among subjects who had changes in bad eating habits. However, the risk of overweight and obesity did not differ among subjects with changes in good eating habits regardless of their sleep quality.

## 1. Introduction

The number of international students enrolled in host countries is gradually increasing worldwide. According to the data released by UNESCO (United Nations Educational, Scientific and Cultural Organization)’s Institute for Statistics, the number of international students was 2 million in 2000 but increased to over 5.3 million worldwide in 2017 [1]. These trends have been shown in South Korea. Compared to a year ago, the number of international students increased by 12.6% to 160,165 persons from 189 countries in 2019 according to statistics from the Ministry of Education for Korea [2].

International students experience environmental and cultural differences in their host country [3,4]. They might have to adapt to the environment or culture of their host [5]. Acculturation to different environments or cultures may have adverse effects on health conditions [6,7]. In other words, international students could experience numerous problems, including academic difficulties, different dietary patterns, and difficulties related to socioeconomic status that could be defined as acculturative stress. Acculturative stress consists of psychological variables and social stress, such as discrimination, homesickness, perceived hate, fear, insecurity, stress due to change or culture shock, and guilt, all of which can result in poor health status [7,8]. In particular, increased stress status is associated with poor sleep [9,10].

Sleep patterns, including sleep duration and quality, and sleep problems are related to health status, such as obesity [11,12,13,14], cardiovascular disease [15], type 2 diabetes [11], and mental health [9]. In particular, it is well-established that poor sleep quality influences dietary quality or energy intake, which affects obesity [13,14]. Our previous study reported that the association of insufficient sleep with an increased risk of obesity is affected by dietary consumption [12]. Additionally, poor sleep quality is associated with an increased dietary consumption, poor eating habits, and more irregular dietary patterns [13,14].

Though the associations of abnormal sleep patterns and eating habits with obesity are well-accepted [12,13,14], it is unclear whether these associations are similar in international students. Scientific research on sleep and dietary patterns among international students is needed to help properly manage the health status of the increasing number of international students. Therefore, this study hypothesized that international students experienced poor sleep quality or changes in eating habits after studying abroad and that these changes were associated with overweight and obesity. To test this hypothesis, we examined sleep quality and changes in eating habits after students had studied abroad and the relationships among sleep quality, eating habits, and overweight and obesity among international students in South Korea.

## 2. Methods

### 2.1. Study Subject Selection

This cross-sectional study examined the associations among sleep quality, changes in eating habits after studying abroad, dietary consumption, and overweight and obesity among international students from October 2019 to January 2020. Before recruiting the subjects, this study was approved by the Institutional Review Board of the Kunsan National University (IRB No. 1040117-201910-HR-018-03), and signatures from subjects on a written informed consent form were obtained before initiating the questionnaire and face-to-face interviews. 

Study subjects were enrolled from international students at universities in the Jeonbook, South Korea. At the time of conducting the survey, subjects were supposed to study in South Korea for at least 6 months and had to currently live in South Korea. This study was conducted in the form of person-to-person interviews using a developed questionnaire. A total of 233 international students (112 men and 121 women) were included in this study.

### 2.2. Measures and Data Collection

The study questionnaires were designed to obtain data on demographic characteristics, health-related variables, anthropometric variables, sleep patterns, and changes in eating habits after studying abroad. The questionnaire used for this study was developed in South Korea and therefore had to be translated into Chinese, English, and Vietnamese versions of the questionnaire. 

Demographic characteristics, such as age, gender, nationality, length of stay in South Korea, academic course, and conversational ability, were collected. When classified by country according to statistics from the Ministry of Education for Korea, Asian students accounted for a large proportion (91.9%). Additionally, all international students who answered the study questionnaire were Asian. Therefore, the subjects’ nationality was classified as “Chinese” or “the others (Vietnamese, Filipino, Mongolian, Cambodian, etc.).” Participants were categorized as “≤ undergraduate school (bachelors’ course) or “≥ graduate school (master’s and doctoral course)” based on their current academic course and “≤ beginner” or “≥moderate” based on their conversational Korean ability. 

As health-related variables, subjects responded to questions about current smoking, alcohol consumption, subjective health status, and acculturative stress. Subjects were categorized as “drinkers” or “nondrinkers” based on their current alcohol consumption status and “smokers” and “nonsmokers” based on their smoking status. Subjective health status was assessed using a 4-point Likert scale, and the subjects were divided into “unhealthy,” including ‘unhealthy’ and ‘general,’ and “healthy,” including ‘healthy’ and ‘very healthy.’ Subjects’ acculturative stress levels were measured using the acculturative stress scale for international students (ASSIS), which was developed by Sandhu and Asrabadi [8]. The ASSIS consists of 36 items with 7 subscales: perceived discrimination, homesickness, perceived hate, fear, culture shock, guilt, and miscellaneous. The overall scores possible on this scale ranged from 36 to 180, with higher scores indicating high acculturative stress.

Body mass index (BMI; kg/m^2^) was calculated based on self-reported body weight in kilograms divided by the square of height in meters, and overweight and obesity were defined in accordance with the definition of obesity in Asian populations proposed by the Asia-Pacific Regional Office of the World Health Organization as a BMI ≥ 23.0 kg/m^2^ [16].

Sleep quality was assessed based on the sleep during the previous month using the Pittsburgh Sleep Quality Index (PSQI) developed by Buysse et al. [17]. This index consists of 7 subscales, including perceived sleep quality, sleep latency, sleep duration, sleep efficiency, sleep disturbances, use of sleeping medication, and daytime dysfunction. The overall possible sleep quality score ranged from 0 to 21, with higher scores indicating poorer sleep quality. The participants were divided into “poor sleeper” or “good sleeper” using a cutoff score of 5.

The questionnaire about changes in international students’ eating habits after studying abroad was modified in accordance with the study by Lee et al. [18]. This questionnaire consists of 7 subscales, including changes in the environment of dietary consumption (10 items), eating problems (10 items), and reasons for unbalanced eating habits (5 items) after studying abroad. Responses of subjects were scored on a 5-point Likert scale, with a score of 0 indicating “strongly disagree” and 10 indicating “strongly agree.” The overall score of changes in eating habits after studying abroad ranged from 0 to 250, with higher scores indicating good eating habits. The Cronbach’s alpha for all 25 items was 0.735.

### 2.3. Statistical Analyses

All continuous variables were assessed for normal distributions before the data were analyzed. The general characteristics according to sleep quality were examined by performing Pearson’s chi-square test or independent t-tests. The generalized linear models were assessed to identify the differences in anthropometrics and changes in eating habits after studying abroad according to sleep quality after adjustment for covariates. Age, gender, nationality, and acculturative stress were included as covariates with potentially confounding effects. The risk of overweight and obesity according to sleep quality after adjusting for covariates was examined using multinomial logistic regression models. After stratification by scores reflecting changes in eating habits after studying abroad, multinomial logistic regression models were used to confirm how the effect of sleep quality on the risk of overweight and obesity was amended by changes in eating habits after studying abroad. A *p*-value of <0.05 was considered significant. All statistical analyses were performed using the SPSS (version 24.0, IBM Corp., Armonk, NY, USA) software for Windows.

## 3. Results

The general characteristics stratified by sleep quality are presented in Table 1. Approximately half of the international students experienced poor sleep quality (47.6%) according to the PSQI. Subjects with good sleep quality were significantly less often Chinese (*p* = 0.029) and graduate students (*p* = 0.010) than those with poor sleep quality. In addition, subjects reporting good sleep quality were healthier (*p* = 0.024) and less likely to be smokers (*p* = 0.016) than those with poor sleep quality. However, no significant differences were observed in gender, age, period after studying abroad, type of residence, conversational Korean ability, acculturative stress, or current alcohol consumption stratified by sleep quality. 

**Figure 1:**
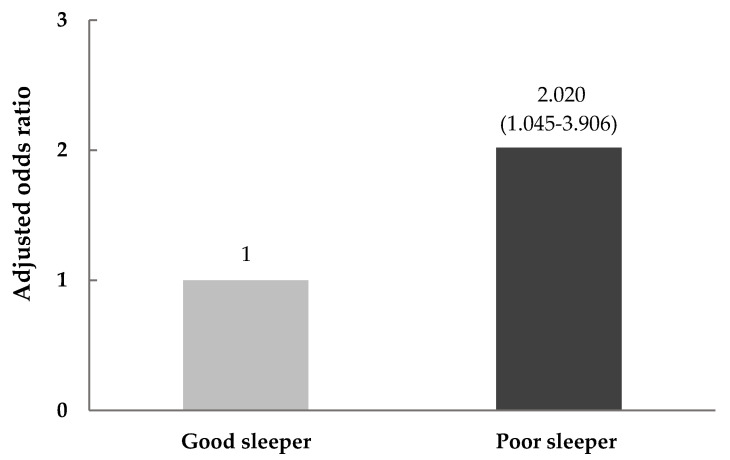
Adjusted odds ratio (OR) for overweight and obesity according to sleep quality. Data are presented as the OR [95% confidence intervals (CI). The OR (95% CI) was calculated in reference to good sleep quality (Pittsburgh Sleep Quality Index (PSQI) ≤ 5) using multinomial logistic regression after adjusting for age, gender, nationality, and acculturative stress.

There were significant differences observed in anthropometrics and the risk of overweight and obesity according to sleep quality (Table 2 and Figure 1). Height (*p* < 0.001) and obesity prevalence (*p* = 0.006) showed differences between good and poor sleepers in the crude model. However, after adjustment for covariates such as age, gender, nationality, and acculturative stress, the good sleepers had a significantly lower BMI values (23.14 ± 7.79 kg/m^2^ vs. 23.95 ± 7.51 kg/m^2^, respectively, *p* = 0.043) and obesity prevalence (18.0% vs. 33.3%, respectively, *p* = 0.037), as well as height (*p* = 0.003) and weight (*p* = 0.008), than poor sleepers (Table 2). Using the subjects with good sleep quality as a reference, the adjusted odds ratio of being overweight and obese for those with poor sleep quality was 2.020-fold (95% confidence interval = 1.045–3.906) higher after controlling for covariates (Figure 1).

The changes in eating habits after studying abroad among international students stratified by sleep quality in the crude and adjusted models are shown Table 3. There were significant differences in changes in eating habits after studying abroad according to sleep quality. The good sleep quality group had a significantly lower total score of changes in eating habits after studying abroad (103.96 ± 28.40 score vs. 120.08 ± 25.93 score, respectively, *p* < 0.001) than the poor sleep quality group. Additionally, subscales of environments of dietary consumption and eating problems after studying abroad were significantly different according to sleep quality (49.59 ± 14.75 score vs. 56.53 ± 13.89 score, respectively, *p* < 0.001 for changes in environments for dietary consumption in the good sleep quality and poor sleep quality groups and 35.12 ± 15.20 score vs. 42.16 ± 17.19 score, respectively, *p* = 0.001 for eating problems), with the exception of the reason for unbalanced eating habits (*p* = 0.165). Among the changes in the environment for dietary consumption, the good sleepers showed lower scores for ‘The number of cooked meals has increased’ (3.03 ± 3.25 score vs. 3.85 ± 3.83 score, respectively, *p* = 0.012), ‘The time to eat has decreased’ (3.46 ± 2.76 score vs. 5.61 ± 2.66 score, respectively, *p* < 0.001), ‘There are difficulties in communicating when buying food’ (2.91 ± 2.75 score vs. 3.73 ± 2.47 score, respectively, *p* = 0.005), ‘I can’t eat what I like’ (4.57 ± 3.11 score vs. 6.13 ± 2.84 score, respectively, *p* = 0.001), and ‘There is an economic problem’ (6.17 ± 2.82 score vs. 7.70 ± 2.29 score, respectively, *p* < 0.001) than the poor sleepers. The subjects with good sleep quality showed lower scores for ‘The irregular hours of work and rest led to irregular meal times’ (4.24 ± 2.93 score vs. 6.73 ± 3.14 score, respectively, *p* < 0.001), ‘I tend to drink heavily’ (1.56 ± 2.26 score vs. 2.93 ± 3.07 score, respectively, *p* < 0.001), and ‘I have increased meat intake’ (3.59 ± 2.82 score vs. 4.35 ± 3.48 score, respectively, *p* = 0.020) among eating problems than those with poor sleep quality. Among the reasons for unbalanced eating habits, only ‘I think this is because I can cook and eat food in my home country, but I can’t eat it in Korea’ (3.61 ± 3.08 score vs. 4.91 ± 3.52 score, respectively, *p* = 0.008) was significantly different between good and poor sleep quality groups. 

**Figure 2:**
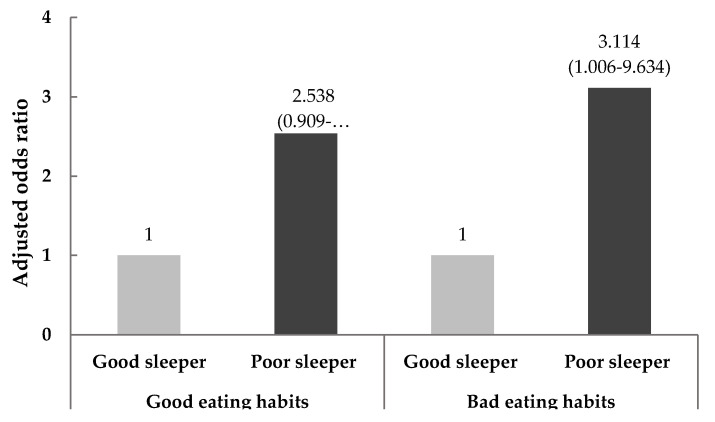
The odds ratios for overweight and obesity according to sleep quality and eating habits after studying abroad. Data are presented as the odds ratios (95% confidence intervals). The ORs (95% CIs) were calculated in reference to good sleep quality (PSQI ≤ 5) using multinomial logistic regression after adjusting for age, gender, nationality, and acculturative stress.

After being stratified into groups according to eating habits after studying abroad, the effect of sleep quality on odds for overweight and obesity was assessed using a multinomial logistic regression analysis after adjusting for covariates, as shown in Figure 2. Among the subjects with bad eating habits, defined as above the median value of eating habits, the adjusted odds ratio for overweight and obesity was 3.114-fold (95% CI = 1.006–9.634) higher in poor sleepers, using the good sleepers as a reference. However, no association was observed between the risk of overweight and obesity according to sleep quality among subjects with good eating habits.

## 4. Discussion

In this study, 47.6% of international students in South Korea reported poor sleep quality according to the criterion of the PSQI [17]. Sleep quality was associated with obesity prevalence, BMI, risk of overweight and obesity, and eating habits after studying abroad according to sleep quality in a model that was adjusted for age, gender, nationality, and acculturative stress. Additionally, when stratified according to the median value of changes in eating habits after studying abroad, a significant association was observed between sleep quality and the adjusted odds ratio of being overweight and obese; among subjects with changes in bad eating habits, subjects with low sleep quality showed an increased trend in the risk of overweight and obesity, whereas subjects with changes in good eating habits did not differ in their risk of overweight and obesity according to sleep quality.

From our results, about half of the international students slept with poor quality. These results were similar to those of previous studies [5,19], which showed that international students, especially Asian students, experienced more sleep problems. Additionally, our results showed that international students with poor quality of sleep showed a higher BMI and adjusted odds ratio of being overweight and obese than those with good quality of sleep. These findings were consistent with previous results [11,12,13,14], which is well-accepted for the relationship between abnormal sleep patterns and obesity. However, this relationship among international students is reported for the first time in this study. 

Most international students change their dietary patterns and eating habits as they experience dietary acculturation, which could potentially have adverse health effects [6]. It is well-established that poor sleep quality could affect appetite or dietary habits, such as dietary quality or total energy consumption, as well as poor eating habits [12,13,14], which is in agreement with our results. In this study, subjects who had poor sleep quality showed negative changes in eating habits after studying abroad compared those who had good sleep quality. 

Interestingly, after being divided into groups according to the changes in eating habits after studying abroad, the risk of overweight and obesity was significantly different according to sleep quality. In particular, subjects with low sleep quality among subjects with bad eating habits showed an increased trend in the risk of overweight and obesity. These findings did not show any trend in regard to sleep quality among subjects with good eating habits. Generally, epidemiologic studies have consistently shown that abnormal sleep patterns could cause obesity through changes in dietary habits [12,20]. One of the explainable mechanisms by which abnormal sleep patterns are associated with increased appetite and dietary consumption is by reducing circulating leptin and elevating ghrelin, which may lead to an increased risk of obesity. However, the proposed mechanism suggested by Chaput [13] based on experimental and clinical studies is that the increased consumption of dietary energy related to abnormal sleep appears to be driven by reinforcing hedonic stimuli rather than changes in appetite hormones such as leptin and ghrelin. In other words, individuals who experience poor sleep patterns show an increased tendency to overeat, especially carbohydrate-rich food, despite a feeling of satiety. Additionally, the association between poor sleep patterns and the consumption of energy-dense food items such as desserts or sweets, which leads to obesity, is mediated by psychological factors such as mood and subjective stress [21]. The psychological problems experienced by international students could deteriorate sleep quality [9,10]. These factors could exacerbate the eating habit-related problems that may occur after studying abroad [6,7] and could lead to obesity in international students. Conversely, these results are in opposition to few studies reported previously [22,23]. Specifically, unhealthy dietary habits such as the consumption of carbohydrate-rich meals with high glycemic indexes or high caloric dietary consumptions affected alterations in the sleep duration and quality. Additionally, Spaeth et al. reported unhealthy body composition or body weight associated with short sleep duration and low sleep quality [24]—though they could not accurately draw the causality of the relationship between sleep pattern, diet, and obesity, the relationship between those could be influenced in both directions. 

This study was the first to demonstrate that poor sleep quality may influence overweight and obesity status in international students, and this association is affected by changes in eating habits after studying abroad. However, those findings should be interpreted carefully because of several limitations. First, the sample size, which was obtained through surveys in a small city in South Korea, was too small. Second, to measure sleep pattern, a PSQI questionnaire, which is currently the most commonly used tool for easily and subjectively measuring sleep quality, was used [17]. In fact, sleep quality measured by the PSQI is weakly related to the method of measuring objective sleep variables such as actigraphy and polysomnography [25,26]. However, the PSQI is significantly related with sleep diaries [25,26], as well psychological and subjective stress symptoms [25,27]. Thus, although the PSQI was appropriate to study for our hypothesis that abnormal sleep pattern could be caused by the acculturative stress that is experienced due to the relocation from their home country location to South Korea, our findings are needed to be clarified using methods for objective sleep quality assessments. Third, all international students were Asian. Fourth, even in the same Asian population, dietary consumption according to ethnic group or nationality vary uniquely, so it is difficult to convert various food consumption patterns into nutrients. Consequently, this study did not provide quantifiable data about amounts or consumption of diet for estimating the dietary quality. Finally, although the definition of overweight and obesity in this study used the definition of obesity in Asian populations [16], the definition of obesity varies by country, even in the same Asian region. In addition, BMI was used as a proxy measure for overweight or obesity in the current study; however, additional studies using direct measures such as waist circumference and body composition are needed. Therefore, these results may not be entirely generalizable to international students from other countries, and future studies are needed to confirm these results in different populations and large sample sizes.

## 5. Conclusions

In summary, our study demonstrated that sleep patterns are related to differences in the risk of overweight and obesity and eating habits, after studying abroad. Additionally, among international students with good eating habits after studying abroad, those with poor sleep quality showed an increasing trend toward overweight and obesity compared with those with good sleep quality. However, this relationship was not found among those with bad eating habits after studying abroad. These findings suggested that healthy status and proper body weight could be maintained by improving and managing sleep patterns and eating habits, which can be used as a strategy to promote and manage the health of the increasing number of international students.

## Figures and Tables

**Table 1:** General characteristics according to sleep quality.

General Characteristics	Good Sleeper (*n* = 122)	Poor Sleeper (*n* = 111)	*p*-Value *
Gender (men)	44.3	52.3	0.138
Age(years)	25.75 ± 5.11	27.12 ± 6.00	0.064
Length of stay in South Korea (months)	25.11 ± 13.68	24.99 ± 16.08	0.953
Nationality (Chinese)	77.0	87.4	0.029
Academic course (≥graduate school course)	50.8	66.7	0.010
Conversational ability (≥moderate)	23.8	22.5	0.473
Subjective health status (healthy)	90.2	80.2	0.024
Acculturative stress	82.55 ± 16.79	86.36 ± 16.88	0.086
Smoking (smoker)	14.8	27.0	0.016
Alcohol consumption (drinker)	75.4	83.8	0.078

Values represent means ± SD or %. * *p* values were calculated using Student’s t-test or *x^2^*-test.

**Table 2:** Anthropometrics and obesity prevalence according to sleep quality. BMI: body mass index.

Anthropometrics and Obesity Prevalence	Good Sleeper (*n* = 122)	Poor Sleeper (*n* = 111)	*p*-Value *	*p*-Value **
Height (cm)	167.14 ± 8.58	170.92 ± 6.88	<0.001	0.003
Weight (kg)	65.55 ± 25.76	70.58 ± 25.08	0.134	0.008
BMI (kg/m^2^)	23.14 ± 7.79	23.95 ± 7.51	0.419	0.043
Overweight and obesity prevalence	18.0	33.3	0.006	

Values represent means ± SD or %. * *p* values were calculated using Student’s t-test or *x^2^*-test. ** *p* values were calculated using a generalized linear model adjusted for age, gender, nationality and acculturative stress.

**Table 3:** Changes in eating habits after studying abroad according to sleep quality.

Changes in eating habits	Good Sleeper (*n* = 122)	Poor Sleeper (*n* = 111)	*p*-Value *	*p*-Value **
I. Change in environment of dietary consumption	49.59 ± 14.75	56.53±13.89	<0.001	<0.001
The number of food purchases has increased.	5.29 ± 3.44	5.41 ± 3.53	0.795	0.814
The number of cooked meals has increased.	3.03 ± 3.25	3.85 ± 3.83	0.081	0.012
The range of food options has been reduced.	5.45 ± 2.86	5.34 ± 3.24	0.778	0.582
There is no one who helps make food.	5.51 ± 3.42	6.46 ± 3.45	0.035	0.129
The time to eat has decreased.	3.46 ± 2.76	5.61 ± 2.66	<0.001	<0.001
There are difficulties in communicating when buying food.	2.91 ± 2.75	3.73 ± 2.47	0.017	0.005
I can’t eat what I like.	4.57 ± 3.11	6.13 ± 2.84	<0.001	0.001
I started to eat mainly Korean food.	6.99 ± 2.27	7.48 ± 2.83	0.149	0.489
There is an economic problem.	6.17 ± 2.82	7.70 ± 2.29	<0.001	<0.001
When choosing food, the influence of a friend is great.	5.39 ± 3.12	5.65 ± 3.78	0.564	0.792
II. Eating problems	35.12 ± 15.20	42.16 ± 17.19	0.001	0.001
The irregular hours of work and rest lead to irregular mealtimes.	4.24 ± 2.93	6.73 ± 3.14	<0.001	<0.001
I can’t eat well because I don’t have time.	3.18 ± 2.88	2.95 ± 3.32	0.581	0.837
I only eat when I’m hungry.	3.44 ± 2.79	3.67 ± 3.68	0.596	0.519
The number of binge eating times depending on the taste of the food has increased.	4.71 ± 3.07	5.36 ± 3.37	0.126	0.198
The number of meals skipped to lose weight has increased.	2.34 ± 2.92	3.00 ± 3.21	0.104	0.172
The number of times breakfast is skipped to binge lunch has increased.	2.85 ± 2.81	3.31 ± 3.07	0.231	0.095
I tend to drink heavily.	1.56 ± 2.26	2.93 ± 3.07	<0.001	<0.001
My vegetable intake has decreased.	3.96 ± 3.03	3.85 ± 2.97	0.793	0.798
My meat intake has increased.	3.59 ± 2.82	4.35 ± 3.48	0.070	0.020
I purchases and eat fast food often.	5.27 ± 3.34	6.01 ± 3.15	0.080	0.128
III. Reason for an unbalanced eating habits	19.24 ± 9.15	21.37 ± 8.60	0.068	0.165
I think it is because I eat alone when I am in Korea, and I eat with family at home.	4.92 ± 2.93	4.23 ± 3.07	0.083	0.071
I think this is because I can buy food easily in my home country, but it is difficult to buy food in Korea.	3.98 ± 2.65	4.55 ± 3.09	0.131	0.216
I think it’s because Korea doesn’t have access to food that I eat every day in my home country.	3.20 ± 2.56	3.60 ± 2.85	0.252	0.265
I think this is because I can cook and eat food in my home country, but I can’t eat the same foods in Korea.	3.61 ± 3.08	4.91 ± 3.52	0.003	0.008
I think it’s mainly because I eat out.	3.55 ± 2.84	4.08 ± 2.90	0.159	0.346
Total score of eating habits	103.96 ± 28.40	120.08 ± 25.93	<0.001	<0.001

Values represent means ± SDs. * *p* values were calculated using Student’s t-test. ** *p* values were calculated using a generalized linear model adjusted for age, gender, nationality, and acculturative stress.

## References

[1] UNESCO Facts and Figures, Mobility in Higher Education. http://data.uis.unesco.org.

[2] Ministry of Education for Korea. https://www.moe.go.kr.

[3] Ogunsanya M.E., Bamgbade B.A., Thach A.V., Sudhapalli P., Rascati K.L. (2018). Determinants of health-related quality of life in international graduate students. Curr. Pharm. Teach. Learn..

[4] Edwards J.S., Hartwell H.L., Brown L. (2010). Changes in food neophobia and dietary habits of international students. J. Hum. Nutr. Diet..

[5] Ul, Haq I., Mariyam Z., Li M., Huang X., Jiang P., Zeb F., Wu X., Feng Q., Zhou M. (2018). A Comparative Study of Nutritional Status, Knowledge Attitude and Practices (KAP) and Dietary Intake between International and Chinese Students in Nanjing, China. Int. J. Environ. Res. Public Health.

[6] Almohanna A., Conforti F., Eigel W., Barbeau W. (2015). Impact of Dietary Acculturation on the Food Habits, Weight, Blood Pressure, and Fasting Blood Glucose Levels of International College Students. J. Am. Coll. Health.

[7] Silva N.D., Dillon F.R., Verdejo T.R., Sanchez M., De La Rosa M. (2017). Acculturative Stress, Psychological Distress, and Religious Coping Among Latina Young Adult Immigrants. Couns. Psychol..

[8] Sandhu D.S., Asrabadi B.R. (1994). Development of an Acculturative Stress Scale for International Students: Preliminary findings. Psychol. Rep..

[9] Oftedal S., Kolt G.S., Holliday E.G., Stamatakis E., Vandelanotte C., Brown W.J., Duncan M.J. (2019). Associations of health-behavior patterns, mental health and self-rated health. Prev. Med..

[10] Geiker N.R.W., Astrup A., Hjorth M.F., Sjödin A., Pijls L., Markus C.R. (2018). Does stress influence sleep patterns, food intake, weight gain, abdominal obesity and weight loss interventions and vice versa?. Obes. Rev..

[11] Reutrakul S., Cauter V.E. (2018). Sleep influences on obesity, insulin resistance, and risk of type 2 diabetes. Metabolism.

[12] Doo M., Kim Y. (2017). The Consumption of Dietary Antioxidant Vitamins Modifies the Risk of Obesity among Korean Men with Short Sleep Duration. Nutrients.

[13] Chaput J.P. (2014). Sleep patterns, diet quality and energy balance. Physiol. Behav..

[14] Frank S., Gonzalez K., Lee-Ang L., Young M.C., Tamez M., Mattei J. (2017). Diet and Sleep Physiology: Public Health and Clinical Implications. Front. Neurol..

[15] St-Onge M.P., Zuraikat F.M. (2019). Reciprocal Roles of Sleep and Diet in Cardiovascular Health: A Review of Recent Evidence and a Potential Mechanism. Curr. Atheroscler. Rep..

[16] World Health Organization (2000). The Asia-Pacific Perspective: Redefining Obesity and Its Treatment.

[17] Buysse D.J., Reynolds C.F., Monk T.H., Berman S.R., Kupfer D.J. (1989). The Pittsburgh Sleep Quality Index: A new instrument for psychiatric practice and research. Psychiatry Res..

[18] Li G. (2013). The Change of Dietary Environment and Snacking Habits of Chinese Students in South Korea. Master’s Thesis.

[19] Tan J.B., Yates S. (2011). Academic expectations as sources of stress in Asian students. Soc. Psychol. Educ..

[20] Tambalis K.D., Panagiotakos D.B., Psarra G., Sidossis L.S. (2018). Insufficient Sleep Duration Is Associated With Dietary Habits, Screen Time, and Obesity in Children. J. Clin. Sleep Med..

[21] Moubarac J.C., Cargo M., Receveur O., Daniel M. (2013). Psychological distress mediates the association between daytime sleepiness and consumption of sweetened products: Cross-sectional findings in a Catholic Middle-Eastern Canadian community. BMJ Open.

[22] Afaghi A., O’Connor H., Chow C.M. (2007). High-glycemic-index carbohydrate meals shorten sleep onset. Am. J. Clin. Nutr..

[23] Crispim C.A., Zimberg I.Z., dos Reis B.G., Diniz R.M., Tufik S., de Mello M.T. (2011). Relationship between food intake and sleep pattern in healthy individuals. J. Clin. Sleep Med..

[24] Spaeth A.M., Dinges D.F., Goel N. (2017). Objective Measurements of Energy Balance Are Associated With Sleep Architecture in Healthy Adults. Sleep.

[25] Buysse D.J., Hall M.L., Strollo P.J., Kamarck T.W., Owens J., Lee L., Reis S.E., Matthews K.A. (2008). Relationships between the Pittsburgh Sleep Quality Index (PSQI), Epworth Sleepiness Scale (ESS), and clinical/polysomnographic measures in a community sample. J. Clin. Sleep Med..

[26] Grandner M.A., Kripke D.F., Yoon I.Y., Youngstedt S.D. (2006). Criterion validity of the Pittsburgh Sleep Quality Index: Investigation in a non-clinical sample. Sleep Biorhythms.

[27] Carpenter J.S., Andrykowski M.A. (1998). Psychometric evaluation of the Pittsburgh Sleep Quality Index. J. Psychosom. Res..

